# Green Fluorescence of *Cytaeis* Hydroids Living in Association with *Nassarius* Gastropods in the Red Sea

**DOI:** 10.1371/journal.pone.0146861

**Published:** 2016-02-03

**Authors:** Andrey A. Prudkovsky, Viatcheslav N. Ivanenko, Mikhail A. Nikitin, Konstantin A. Lukyanov, Anna Belousova, James D. Reimer, Michael L. Berumen

**Affiliations:** 1 Department of Invertebrate Zoology, Biological Faculty, Lomonosov Moscow State University, Moscow, Russia; 2 A.N. Belozersky Institute of Physico-chemical Biology, Lomonosov Moscow State University, Moscow, Russia; 3 Institute of Bioorganic Chemistry, Moscow, Russia; 4 Molecular Invertebrate Systematics and Ecology Laboratory, Department of Biology, Chemistry, and Marine Sciences, Faculty of Science, University of the Ryukyus, Okinawa, Japan; 5 Tropical Biosphere Research Center, University of the Ryukyus, Okinawa, Japan; 6 Red Sea Research Center, Division of Biological and Environmental Science and Engineering, King Abdullah University of Science and Technology, Thuwal, Saudi Arabia; University of Minnesota, UNITED STATES

## Abstract

Green Fluorescent Proteins (GFPs) have been reported from a wide diversity of medusae, but only a few observations of green fluorescence have been reported for hydroid colonies. In this study, we report on fluorescence displayed by hydroid polyps of the genus *Cytaeis* Eschscholtz, 1829 (Hydrozoa: Anthoathecata: Filifera) found at night time in the southern Red Sea (Saudi Arabia) living on shells of the gastropod *Nassarius margaritifer* (Dunker, 1847) (Neogastropoda: Buccinoidea: Nassariidae). We examined the fluorescence of these polyps and compare with previously reported data. Intensive green fluorescence with a spectral peak at 518 nm was detected in the hypostome of the *Cytaeis* polyps, unlike in previous reports that reported fluorescence either in the basal parts of polyps or in other locations on hydroid colonies. These results suggest that fluorescence may be widespread not only in medusae, but also in polyps, and also suggests that the patterns of fluorescence localization can vary in closely related species. The fluorescence of polyps may be potentially useful for field identification of cryptic species and study of geographical distributions of such hydroids and their hosts.

## Introduction

Fluorescent proteins (FPs) related to the Green Fluorescent Protein (GFP) family have been reported in many taxa of marine organisms, including Cnidaria (Hydrozoa and Anthozoa), Ctenophora, Arthropoda, and Cephalochordata [1, 2]. Artificially engineered FPs have a broad range of excitation and emission spectra and are used for *in vivo* fluorescence labeling in a variety of different cellular systems and transgenic organisms [2, 3]. Progress in FP technologies has arisen from the discovery of novel natural FPs with unusual properties.

Within Hydrozoa, fluorescence has been detected in the hydromedusae of a diverse range of hydrozoans [4–18]. Green fluorescence (GF) has been noted in the medusae of at least 12 families of hydrozoans [11–14, 19]. Fluorescence has been detected in the subumbrella, umbrellar margin, radial canals, manubrium, tentacles or tentacular bulbs of medusa, often with varying patterns between species, genera, or families.

However, fluorescence has only been reported from the hydroids of six species, the majority within the family Campanulariidae (*Clytia hemisphaerica*, *Obelia geniculata*, *O*. *longissima*, *O*. *dichotoma*, and *O*. *bidentata*). Campanulariid hydroids have fluorescence in their photocytes [5]. Photocytes with GF have been detected in the upright shoots of *O*. *geniculata* (Linnaeus, 1758), and GF is concentrated in the tip of the pedicel of *O*. *bidentata* Clark, 1875 [9]. Recently, a presumably species-specific fluorescence pattern (dispersed spots of fluorescence) was noted in the hypostome of *O*. *dichotoma* (Linnaeus, 1758) [20]. Additionally, oval-shaped spots of GF have been detected in the middle part of the hydrocaulus of *Clytia* [14].

The only other hydroid in which fluorescence has been detected is *Cytaeis uchidae* Rees, 1962 within the family Cytaeididae. In *C*. *uchidae* from Japan, GF was found in the epithelium below the tentacles [17]. Additionally, the medusae of *C*. *uchidae* have fluorescence in the epithelium of the exumbrella and subumbrella as well as in the gonads [17].

During a recent biodiversity survey in the southern Red Sea, several *Cytaeis*-like hydroid colonies displaying fluorescence were collected. The hydroids were epibiotic on small gastropod molluscs. In this study, we examined the fluorescence patterns of these hydroid specimens and compared the observed patterns of distribution and fluorescence with past literature in order to ascertain if such fluorescence patterns are species-specific. Species identification of hydroid colonies remains problematic, and species-specific fluorescence patterns may be a useful diagnostic tool in field identification and taxonomy.

## Material and Methods

### Ethics statement

This research was carried out under the general auspices of King Abdullah University of Science and Technology’s (KAUST) arrangements for marine research with the Saudi Arabian Coast Guard and the Saudi Arabian Presidency of Meteorology and Environment. These are the relevant Saudi Arabian authorities governing all sea-going research actions in the Saudi marine environment. KAUST has negotiated a general and broad permission for marine research in Saudi Arabian Red Sea waters with these two agencies and thus there is no permit number to provide. The animal use protocol was performed in accordance with Bioethics Committee of Lomonosov Moscow State University and KAUST’s Institutional Biosafety and BioEthics Committee (at present, the committees do not provide a specific approval numbers for non-vertebrate animal research).

### Specimen collection, morphological examination

**Fig 1:**
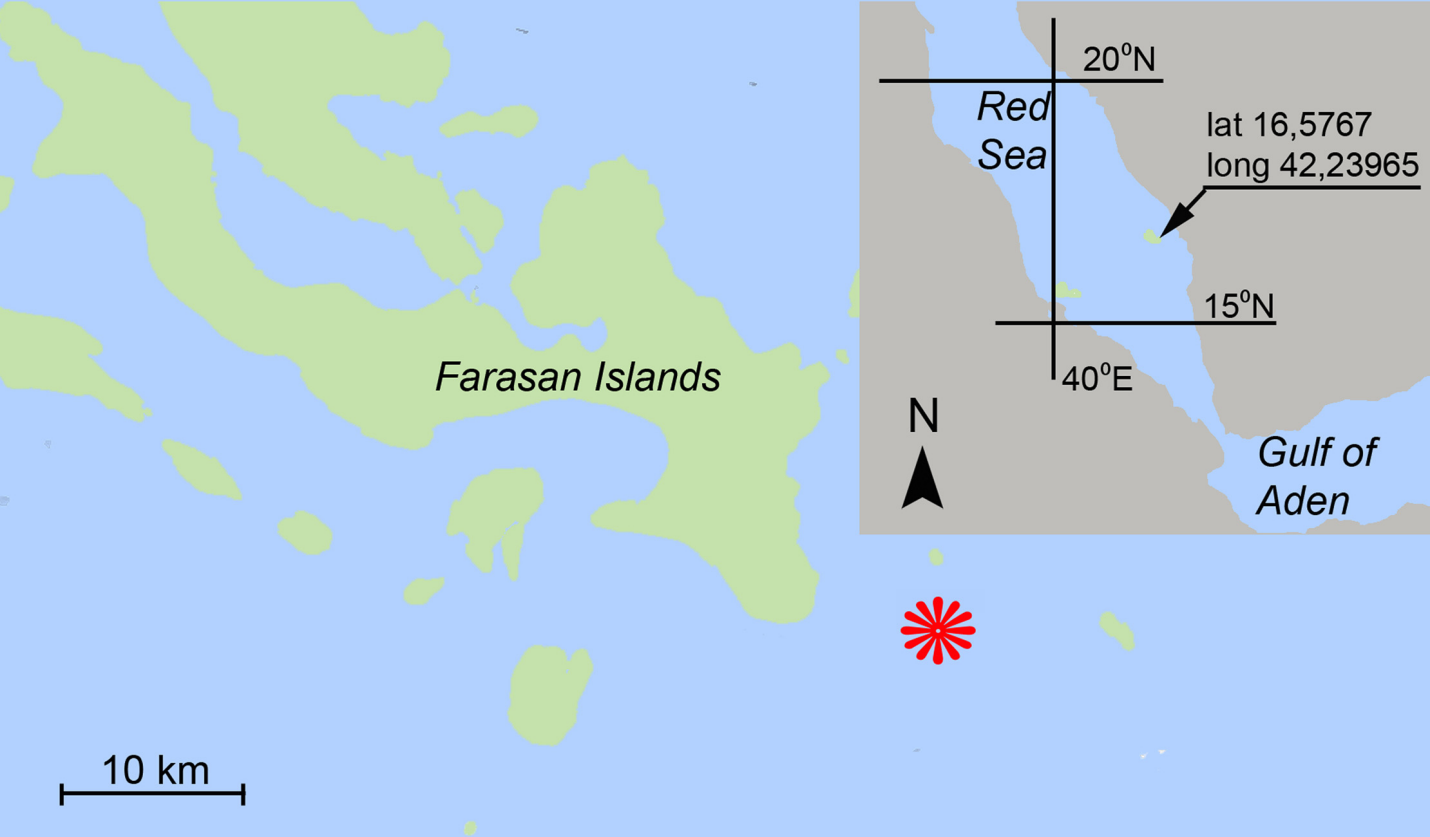
The sampling locality and study area in the Farasan Islands (Saudi Arabia), a complex of islands (indicated in green) in the southern Red Sea (inset). The red star indicates sampling locality within the Farasan Islands group. (The base geographic layer was downloaded from the Landsat 8 satellite database (http://libra.developmentseed.org, Accessed 13 October 2015), via CC by 3.0 (https://creativecommons.org/licenses/by/3.0/)).

A total of 32 specimens of *Nassarius margaritifer* (Dunker, 1847) (Neogastropoda: Buccinoidea: Nassariidae) identified by Alexander Fedosov (A.N. Severtsov Institute of Ecology and Evolution, Moscow, Russia) were collected from the southern Red Sea during night dive at Hindiya Reef, Farasan Islands, Saudi Arabia (latitude 16.5767, longitude 42.23965), on October 26, 2014 (19.00–22.00), from depths of 3–8 m, water temperature 30°C (Fig 1). The gastropods were on the surface of the sandy bottom, actively burying into the sand in response to the blue light of an underwater flashlight.

The colonies of hydroid polyps were attached to the outer shell surfaces of actively moving *N*. *margaritifer* and were observed underwater and aboard the research vessel immediately after collection in petri dishes. 47 polyps were additionally observed under a Leica MZ6 stereomicroscope. Photographs were taken with a Canon 550D camera and MP-E macro lens.

The fluorescence of polyps was observed and photographed at nighttime underwater (*in situ*) and aboard the research vessel by means of a UV torch NIGHTSEA FL-1 dive light with a yellow filter. Collected specimens of *N*. *margaritifer* with hydroids attached were then preserved in 96% ethanol (to avoid fast degradation of fluorescence signal and DNA stored in -20°C) or 10% salt water formalin; 2 specimens of *N*. *margaritifer* with attached hydroids preserved in 96% ethanol are deposited in the Zoological Museum of Lomonosov Moscow State University (catalog number Ea-169).

**Fig 2:**
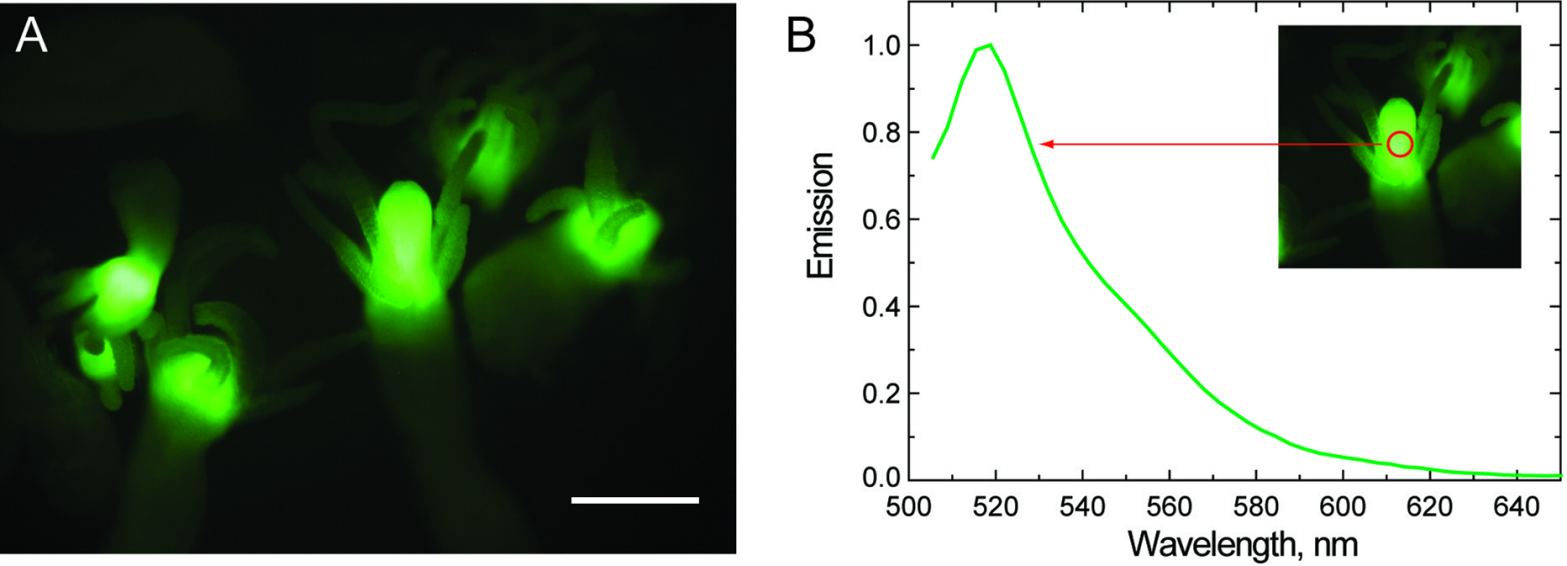
Microscopy imagery of fluorescence of hydroids (*Cytaeis* sp.) collected in the Saudi Arabian Red Sea. (A) green fluorescence of the polyps, scale bar 0.3 mm; (B) emission spectrum measured directly from fluorescent part of the polyp (red circle in the inset).

Fluorescence images of 52 polyps were also taken using a SZX12 (Olympus, Japan) stereomicroscope with GFP filter set (excitation at 460–490 nm, emission at 520–550 nm). Emission spectra of specimens were measured directly from a fluorescent portion of the hydroid polyp using the same stereomicroscope equipped with a SMS 2 VIS (Pannhoff Optische Messtechnik, Germany) spectrophotometer, which can record spectra from a small area within the field of view (shown in Fig 2B).

### Molecular identification

DNA was extracted from a total of two ethanol-preserved hydroid specimens using a Diatom DNA Prep 100 Kit (Isogene, Moscow, Russia). Fragments of nuclear (18S ribosomal DNA, internal transcribed spacer region (ITS1+5.8S+ITS2), 28S ribosomal DNA) and mitochondrial (cytochrome oxidase I (COI), 16S ribosomal DNA) were amplified using an Encyclo Plus PCR kit (Evrogen, Moscow, Russia) and specific primers [21–25]. The following PCR conditions were used: 3 min at 95°C, 37 cycles of 94°C for 20s, followed by annealing at respective temperatures for 30s, 72°C for 1 min 30s, and then a final elongation at 72°C for 5 min. PCR products were purified with preparative electrophoresis in 1% agarose gel and DNA bands of appropriate length were excised from the gels and extracted using a GelPrep spin-column kit (Cytokine, Saint Petersburg, Russia). Extracted DNA was sequenced on an ABI 3730 capillary sequencer in both directions.

The sequences were assembled and edited using MEGA 6.0 [26]. Sequences obtained in this study were deposited in GenBank (Accession Numbers KR233812–KR233813, KT326191) and compared for similarity with previous hydroid sequences using the National Center for Biotechnology Information’s Basic Local Alignment Search Tool (NCBI BLAST). Sequences were compared only by similarity (i.e., DNA barcoding [27]) to identify specimens; no further phylogenetic analyses were performed.

## Results

### Morphology and Fluorescence

**Fig 3:**
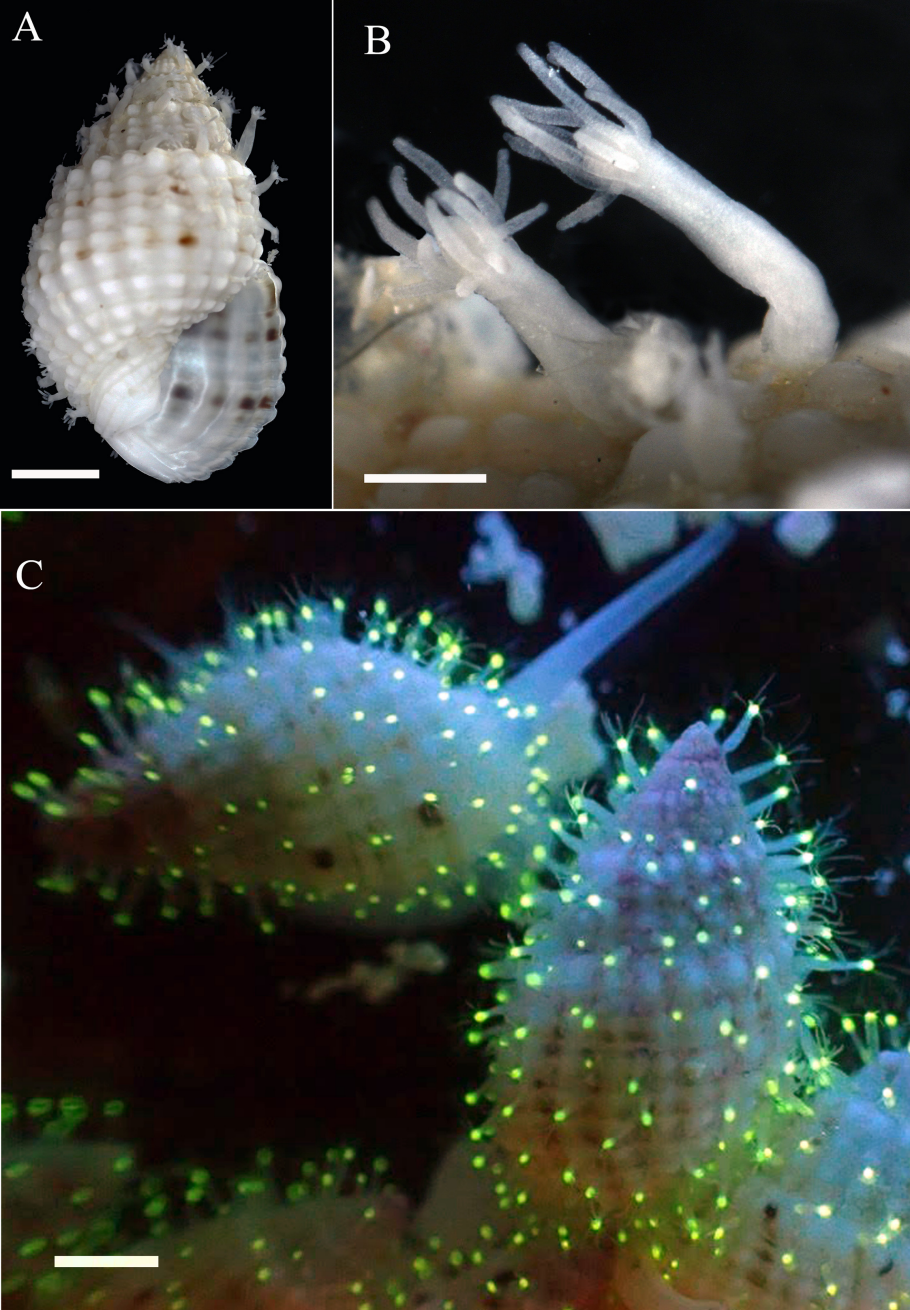
Hydroid polyps of *Cytaeis* sp. from the Saudi Arabian Red Sea, scale bar 2 mm; (A) fluorescence of living polyps on the shell of the gastropod *Nassarius margaritifer*; (B) polyps on the shell of a *N*. *margaritifer* specimen, scale bar 2 mm; (C) close-up of polyps, scale bar 0.5 mm.

Hydroid polyps formed dense, creeping, non-polymorphic colonies with ramified stolons, with up to 36 polyps/cm^2^ on the exposed surfaces of *N*. *margaritifer* shells (Fig 3A and 3C). Stolons were in furrows of shells (Fig 3A). Polyps were sessile without distinct pedicel (Fig 3B). Length of five largest polyps was 1.4–1.5 mm. Membranous perisarc cups at the bases of polyps were not found. Bodies of polyps were elongated with diameter about 1/6 of height. The conical hypostome was encircled by 4–14 filiform amphicoronate tentacles with nematocysts. Undischarged nematocysts were 8–9 μm long and 4–5 μm wide (microbasic euryteles) or 6–7 μm long and 4–5 μm wide (desmonemes) (S1 Fig). Medusa buds or gonophores were not found.

Alive and preserved specimens have same patterns and color in situ and lab, and bright green fluorescence was observed in the hypostome of all polyps (Figs 2A & 3C). The emission spectrum measured directly from the hypostome of seven polyps The spectra have been collected at the same time and on different shells had a peak at 518 nm (Fig 2A and 2B). Variability or anatomical differences in expression of green fluorescence was not found. All studied specimens of hydroids of different sizes and from different shells expressed strong signal of the same fluorescence, which was always in the hypostome as shown.

### Genetic analyses

**Fig 4:**
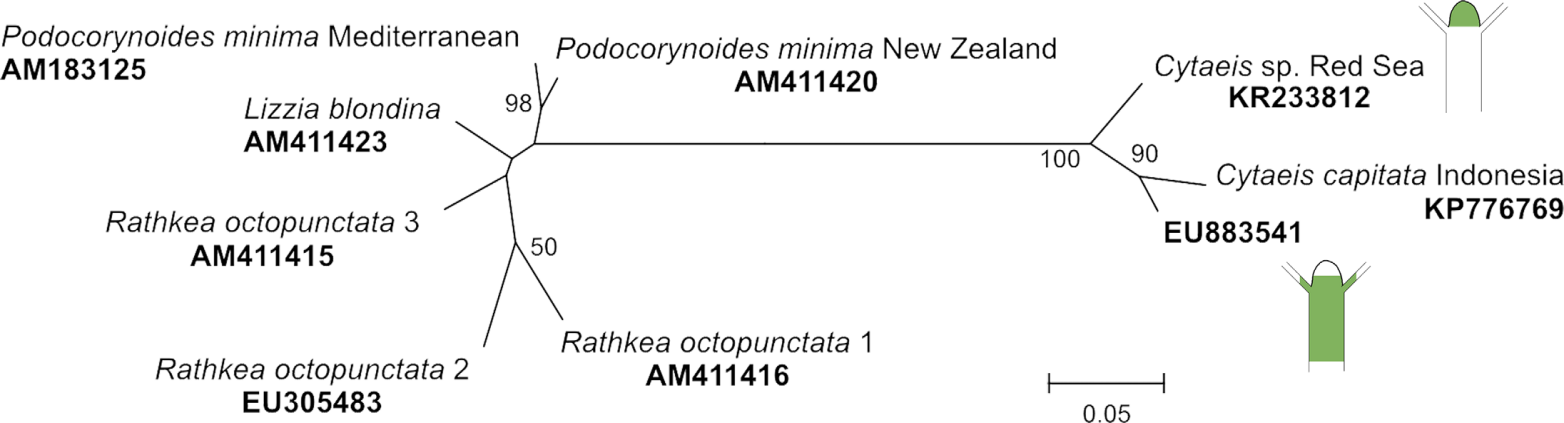
Maximum likelihood tree of 16S mitochondrial rRNA gene sequences from *Cytaeis*, *Podocorynoides*, and their closest relatives (BLAST similarity >90%). *P*. *minima* from Mediterranean and New Zealand clusters within other representatives of the family Rathkeidae (*Rathkea* and *Lizzia*). The Red Sea *Cytaeis* sp clusters with the sequence of *Cytaeis* sp. (EU883541) from Japan and *Cytaeis capitata* from Indonesia. Schematic images of the polyps of *Cytaeis* sp. from Japan and the Red Sea indicate location of the fluorescence.

All but one of 1730 base pairs of 18S rDNA sequences from hydroid specimens were identical to previously reported sequences of *Cytaeis uchidae* and *Podocorynoides minima*, both from Japan (GenBank Accession Numbers JQ407405 and EU883546, respectively). The acquired 28S rRNA sequence was 1050 bp in length and 99% (1041/1050 bp) identical to a previously reported sequence from *P*. *minima* (EU883552). The acquired 28S rRNA sequence was identical to a previously reported 253 bp 28S rDNA fragment of *C*. *uchidae* (JQ410764). The 100% similarity of ribosomal RNA sequences between *C*. *uchidae* (Cytaedidae) and *P*. *minima* (Rathkeidae), species belonging to different families, was unexpected and suggests either a mislabeling of data or misidentification of specimens. The numbers of available hydrozoan COI sequences in GenBank are limited, and there are no sequences designated as ‘*Cytaeis’* or ‘*Podocorynoides’*. Our obtained COI sequence was most similar (86%) to sequences reported from *Nemopsis bachei* (KC440111, KC440112, KC440115). However, three 16S mitochondrial rDNA sequences of *P*. *minima* and one of *Cytaeis capitata* are present in GenBank, and the novel 16S rRNA sequences from the Red Sea specimens were 92% similar to a sequence of *P*. *minima* from Japan *(*EU883541) and 91% similar to a sequence of *Cytaeis capitata* from Indonesia (KP776769), but only 77% similar to two other 16S rDNA sequences identified as *P*. *minima* from New Zealand and the Mediterranean (AM183125, AM411420, respectively). In the cladistic tree (Fig 4), 16S sequences of *P*. *minima* from New Zealand and Mediterranean fell within a well-supported clade including *Lizzia (*GenBank Accession Number AM411423) and *Rathkea* 1–3 (GenBank Accession Numbers AM411416, EU3055483, AM411416, respectively), while the third sequence of *P*. *minima* from Japan grouped with *C*. *capitata* and our Red Sea specimen. The grouping of *P*. *minima* with *Lizzia* and *Rathkea*, members of the Rathkeidae family, is consistent with current taxonomy of filiferan hydrozoans [28]. Therefore, we consider that the 16S rRNA sequence EU883541 from a hydrozoan from Japan has been misidentified, as the nuclear rRNA sequences from the same hydrozoan (EU883546, EU883552) [29] are 100% similar with sequences of *C*. *uchidae* (JQ407405, JQ410764) [30]. Therefore, although initially confusing, our molecular data confirm the morphological identification of the Red Sea hydrozoan as a representative of the genus *Cytaeis*.

## Discussion

The identification of species of genus *Cytaeis* is based on features (size of polyps, numbers of tentacles, and presence of peridermal cap) that are often fragmentarily present, or present only depending on development stage or physiological state of the colony (S1 Table). With the lack of reliable morphological characters to distinguish hydroids of *Cytaeis*, our findings of intense fluorescence in the hypostome of specimens in this study that are different from other congeners suggests this fluorescence characteristic may be useful feature for species related diagnoses.

Two species of *Cytaeis* (*C*. *nassa* (Millard, 1959)) and *C*. *tetrastyla* (Eschscholtz, 1829) have been reported from the Red Sea [31]. The medusa of *C*. *tetrastyla* (hydroid form still unknown) has been reported circumglobally in the tropics and subtropics [28, 32–33] and may represent a complex of species [34–35]. The hydroid *Cytaeis nassa* with known medusae buds but an unknown medusa has been found in the Red Sea on shells of the gastropods *Nassarius arcularia* (Linnaeus, 1758) and *N*. *fenistratus* (Marrat, 1877). The species was also reported on shells of *N*. *fenistratus* and *N*. *coronatus* (Bruguière, 1789) from South Africa (Inhaca Island) and on *N*. *arcularia* and *N*. *albescens* (Dunker, 1846) from Madagascar [36]. The polyps of *Cytaeis* in this study and those observed before at the Red Sea, as well as the medusa of *C*. *tetrastyla*, may represent records of either the same species or a complex of cryptic species. This taxonomic uncertainty combined with the large number of records of *Cytaeis* associated with *Nassarius* from different localities (S2 Table) together with our molecular results lead us to identify these Red Sea specimens as *Cytaeis* sp.

Hydrozoan species typically have FPs with maximum emission at 495–510 nm [6, 9, 10, 37, 38]. In addition, cyan, yellow, and orange FPs and non-fluorescent purple chromoprotein have been isolated from *Obelia* and *Phialidium* (*Clytia*) species and an unidentified jellyfish (Anthoathecata) [6, 10, 15, 16, 38]. Here, we found that the FP in *Cytaeis* sp. from the Red Sea had a maximum emission at 518 nm. Similar green FPs with slightly red-shifted spectra (emission peak at 515–525 nm) have been characterized in anthozoan species, such as *Acropora eurostoma*, *Echinophyllia echinata*, and *Favites abdita* [39]. Thus, our finding of slightly red-shifted emission spectra in *Cytaeis* sp. can be considered unusual but not unprecedented. While it is not outside the range of expected emission, it may be a species-specific character within the genus *Cytaeis*.

Green fluorescent proteins (GFPs) have often been noted in the medusa stage of hydroids but there have also been several reports of GFPs in early development stages (eggs or planulae) as well as in hydroid colonies. Fluorescence in *Obelia* colonies is localized in the upright parts: in the hydrocaulus, the tip of pedicels, or in polyps [9, 20]. In *Clytia hemisphaerica*, fluorescence is localized to the endoderm of the stolon, the hydranth, and polyp tentacles [18]. From the transcriptome of *C*. *hemisphaerica*, four GFPs with distinct stage- and tissue-specific expression profiles were identified [18]. Fluorescence of the manubrium and gonad tissue showed a fluorescence emission peak at 508–510 nm, whereas in the eggs and tentacle bulbs the emission peaked at 500–502 nm. In other *Phialidium* (*Clytia*) species fluorescence was not detected in the planula stage but green fluorescent cells appeared after metamorphosis in the peduncle at the base of the stalk [7].

Fluorescence has also been seen in anthoathecata hydroids in the congeneric hydroid *Cytaeis uchidae* from Japan [17]. The fluorescence of *C*. *uchidae* is localized in different parts of polyps when compared to our results from *Cytaeis* sp. from the Red Sea. Thus, based on past literature and the results of this study, it appears that even within congeners, fluorescence can be localized within different areas of hydroids [9, 17, 20]. Whether this character is species-specific or varies within the natural ranges of species remains to be seen, but as accurate identification of hydroid polyps is currently very difficult due to lack of morphology characters, the utility of fluorescence localization needs to be examined in more detail.

Associations between hydroids and molluscs have long been recognized (reviewed in [40]), although few studies have investigated the ecological significance of these symbioses (e.g., [41]). The ecological implications of the symbioses between *Nassarius* snails and *Cytaeis* hydroids have been studied [42] but the ecological role of the fluorescence in *Cytaeis* remains poorly understood. Fluorescence in the hypostome of *Cytaeis* sp. has probable ecological significance as prey are likely to be attracted to the tentacles and mouth of the polyps [43]. If this is the case, the fluorescence of polyps could be utilized in sampling and study of nocturnal activity as well as in elucidating the distribution of the hydroids and their hosts. Fluorescence utilized to attract prey to hydroids living on nocturnal hosts needs moonlight for stimulation of fluorescence. The sunlight during sunset or sunrise can be another source of light. However, many aspects of this association remain understudied, including; diurnal/nocturnal activity of the *Nassarius* snails and *Cytaeis* hydroids, host specificity, and intra- and interspecies variation of fluorescence of hydroids at different developmental stages. Finally, the physiological state of *Cytaeis* hydroids and environmental conditions during which fluorescence is observed also require additional study.

## Supporting Information

S1 Fig (a) cnidocysts of *Cytaeis* sp. a. undischarged cnidocysts in a tentacle of a polyp; (b) discharged eurytele from a tentacle of a polyp; (c) discharged desmoneme from a tentacle of a polyp. Scale bar 20 μm (applicable to all images).
(TIF)

S1 Table Species-specific morphological features of hydroid polyps of genus *Cytaeis*. (DOCX)

S2 Table Records from previous literature of genus *Cytaeis* associations with shells of genus *Nassarius*. (DOCX)
