# Supplementary figures and images for: Green Fluorescence of Cytaeis Hydroids Living in Association with Nassarius Gastropods in the Red Sea
Source: PLoS One. 2016 Feb 3;11(2):e0146861. doi: 10.1371/journal.pone.0146861 (PMC4739711; doi:10.1371/journal.pone.0146861)

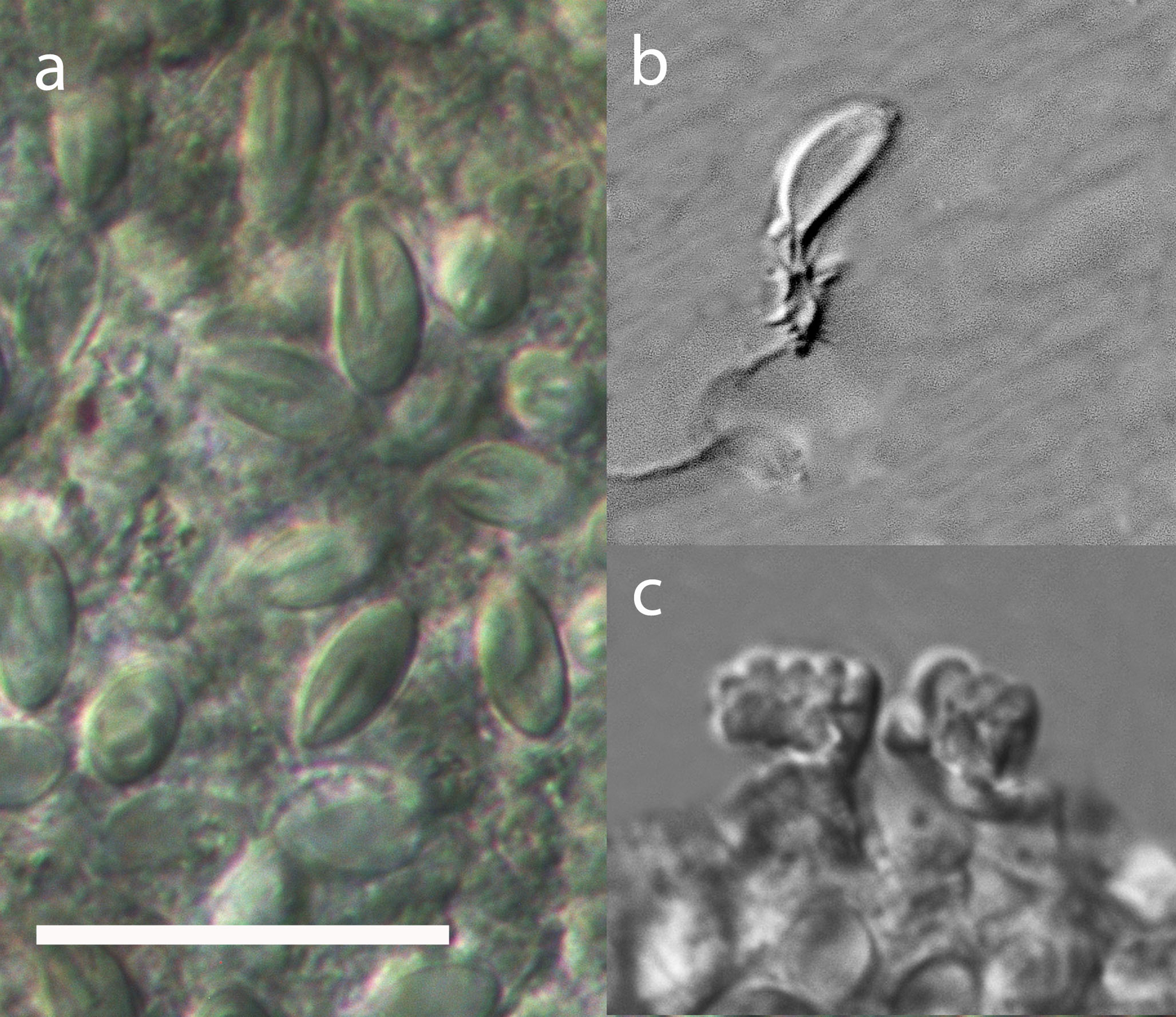

Supplement: S1 Fig — Scale bar 20 μm (applicable to all images). (TIF) [file pone.0146861.s001.tif]
