# Supplementary material for: Green Fluorescence of Cytaeis Hydroids Living in Association with Nassarius Gastropods in the Red Sea
Source: PLoS One. 2016 Feb 3;11(2):e0146861. doi: 10.1371/journal.pone.0146861 (PMC4739711; doi:10.1371/journal.pone.0146861)
Supplement: S1 Table — (DOCX) [file pone.0146861.s002.docx]

**S1 Table.** Species-specific morphological features of hydroid polyps of genus *Cytaeis* (hydroid polyps of *C.adherence,* *C.pusilla*, *C. tetrastyla,* and *C.vulgaris* are unknown).

| **nn** | **Species / References** | **Location** | **Maximum size (mm)** | **Tentacle number** | **Perisarc cup** | **Nematocysts sizes (um)** |
| --- | --- | --- | --- | --- | --- | --- |
|  | *C. niotha* (Pennycuic, 1959) | Australia | 3 | ?-15 | – | – |
|  | *C. niotha* | Australia | – | 14-16 | – | – |
|  | *C. uchidae* Rees, 1962 | Japan | 4 | 6-10 | Present | – |
|  | *C. imperialis* Uchida, 1964 | Japan | 1 | 4-8 | Absent | – |
|  | *C. nuda* Rees, 1962 | Japan | 0.5 | 6-8 | Absent | – |
|  | *C. indica* (Stechow, 1920) | Christmas Island | – | – | Present | – |
|  | *C. nassa* (Millard, 1959) | East and South Africa | 2.5 | 8-16 | Present | 6.3x3.6  8.1x3.1 |
|  | *Cytaeis* sp. (Calder, 1988) | Bermuda | 0.6 | 4-5 | Absent | 5-5.8x3.3-3.8  7.4-8.3x3.5-3.8 |
|  | *C. capitata* Puce et al., 2004 | Indonesia | 1 | 6-12 | Present | 5-5.5x4.5-5,5  7-7.5x5-5.5  18-21x7-9 |
|  | *Cytaeis* sp. (present paper) | The Red Sea | 1.5 | 4-14 | Absent | 6-7x4-5  8-9x4-5 |
