# Supplementary material for: Green Fluorescence of Cytaeis Hydroids Living in Association with Nassarius Gastropods in the Red Sea
Source: PLoS One. 2016 Feb 3;11(2):e0146861. doi: 10.1371/journal.pone.0146861 (PMC4739711; doi:10.1371/journal.pone.0146861)
Supplement: S2 Table — (DOCX) [file pone.0146861.s003.docx]

**S2 Table.** Records from previous literature of genus *Cytaeis* associations with shells of genus *Nassarius.*

| **nn** | ***Nassarius* species** | ***Cytaeis*** **species** | **Locality** | **References** |
| --- | --- | --- | --- | --- |
|  | *N. albescens (*as *Niotha albescens)* | *Cytaeis niotha* | Australia | Rees, 1962 |
|  | *N. albescens (*as *Nassa albescens)* | *Cytaeis nassa* | Madagascar | Millard, 1975 |
|  | *N. arcularia (*as *Nassa arcularia)* | *Cytaeis nassa* | Red Sea, Madagascar | Vervoort, 1967  Millard, 1975 |
|  | *N. caelatus(*as *Zeuxis coelatus)* | *Cytaeis imperialis* | Japan | Uchida, 1964  Hirohito, 1988 |
|  | *N. coronatus (*as *Nassa coronata)* | *Cytaeis nassa* | East and South Africa | Rees, 1962  Millard, 1975 |
|  | *N. fenistratus (*as *Nassa fenestrata)* | *Cytaeis nassa* | Red Sea, East and South Africa | Rees, 1962  Vervoort, 1967  Millard, 1975 |
|  | *N. festivus (*as *Reticunassa festiva, Hinia festivus)* | *Cytaeis uchida* | Japan | Uchida, 1964  Hirohito, 1988 |
|  | *N. globosus (*as *Pliarcularia globosus)* | *Cytaeis capitata* | Indonesia | Puce et al., 2004 |
|  | *N. livescens (*as *Niotha livescens)* | *Cytaeis uchida* | Japan | Rees, 1962  Uchida, 1964  Hirohito, 1988  Hirai, Kakinuma, 1973 |
|  | *N. margaritifer* | *Cytaeis* sp. | Red Sea |  |
